# Ontology-Based High-Level Context Inference for Human Behavior Identification

**DOI:** 10.3390/s16101617

**Published:** 2016-09-29

**Authors:** Claudia Villalonga, Muhammad Asif Razzaq, Wajahat Ali Khan, Hector Pomares, Ignacio Rojas, Sungyoung Lee, Oresti Banos

**Affiliations:** 1 Ubiquitous Computing Lab, Department of Computer Engineering, Kyung Hee University, 1 Seocheon-dong, Giheung-gu, Yongin-si, Gyeonggi-do 446-701, Korea; cvillalonga@oslab.khu.ac.kr (C.V.); asif.razzaq@oslab.khu.ac.kr (M.A.R.); wajahat.alikhan@oslab.khu.ac.kr (W.A.K.); oresti@oslab.khu.ac.kr (O.B.); 2 Department of Computer Architecture and Computer Technology, Research Center for Information and Communications Technologies—University of Granada (CITIC-UGR), C/Periodista Rafael Gomez Montero 2, Granada 18071, Spain; hector@ugr.es (H.P.); irojas@ugr.es (I.R.); 3 Telemedicine Group, Center for Telematics and Information Technology, University of Twente, 7500 AE Enschede, The Netherlands

**Keywords:** context recognition, context inference, ontologies, ontological reasoning, human behavior identification, activities, locations, emotions

## Abstract

Recent years have witnessed a huge progress in the automatic identification of individual primitives of human behavior, such as activities or locations. However, the complex nature of human behavior demands more abstract contextual information for its analysis. This work presents an ontology-based method that combines low-level primitives of behavior, namely activity, locations and emotions, unprecedented to date, to intelligently derive more meaningful high-level context information. The paper contributes with a new open ontology describing both low-level and high-level context information, as well as their relationships. Furthermore, a framework building on the developed ontology and reasoning models is presented and evaluated. The proposed method proves to be robust while identifying high-level contexts even in the event of erroneously-detected low-level contexts. Despite reasonable inference times being obtained for a relevant set of users and instances, additional work is required to scale to long-term scenarios with a large number of users.

## 1. Introduction

A revolutionary wave of smart systems has recently emerged to enable the automatic identification of human behavior. Proposed solutions are generally intended to recognize the primal dimensions of human behavior and context, including people’s activities, emotions and locations. Video systems [1] and on-body sensors [2] have extensively been considered for the recognition of physical activity. Other works have used positioning technologies to track the user location and derive movement patterns [3]. Video, audio or a combination of both modalities have also been proposed for analyzing speech and facial expressions in order to recognize some emotional states [4,5]. Data-driven, knowledge-driven and hybrid methods are normally considered for processing these sensory data to identify human behavior. In data-driven approaches, machine learning techniques are used to detect patterns matching some known contexts describing behavior [6,7,8]. In knowledge-driven approaches, ontologies and rules are utilized to model and infer different contexts [9,10,11]. Both data-driven and knowledge-driven techniques are further combined in hybrid methods to determine various components of human behavior [12,13].

Despite the value of the contributions made for the automatic identification of human behavior, it is fair to say that most existing solutions tend to apply to a sole dimension of behavior. In other words, most systems are only capable of identifying activities, locations or emotions, but generally not a combination of them. While these primitives could be considered in isolation for a preliminary analysis of a person’s behavior, their appropriate combination can lead to more meaningful and richer expressions of context for behavior understanding. Hence, there is a clear opportunity for developing new methods for the automatic identification of richer human behavior information.

This work presents an ontology-based method to intelligently combine cross-domain behavior primitives, also referred to as low-level contexts, in order to infer more abstract human context representations, hereafter high-level contexts. The proposed method particularly extends beyond the state-of-the-art while uniting emotion information as a novel behavioral component together with activity and location data to derive more meaningful contextual information. This paper contributes with an open ontology, the so-called Mining Minds Context Ontology, integrating all of the contextual definitions to link both low and high-level context domains. This work further contributes with the design, implementation and evaluation of a framework, namely the Mining Minds High-Level Context Architecture, which builds on the Mining Minds Context Ontology and reasoning techniques to enable the inference of high-level context from low-level context primitives in real time. The Mining Minds High-Level Context Architecture constitutes the core engine for the inference of high-level behavioral information in the Mining Minds platform [14,15]. Despite the proposed framework being originally devised to serve this platform, the Mining Minds High-Level Context Architecture has been defined in a way so it can be used independently for determining high-level context information from other similar sources of low-level context data.

The rest of the paper is organized as follows. Section 2 presents the related work. Section 3 describes the Mining Minds Context Ontology and presents some examples of context to illustrate the different modeling principles and the inference logic. Section 4 presents the Mining Minds High-Level Context Architecture and describes the inference method for the identification of the user context based on the Mining Minds Context Ontology. Section 5 describes the implementation of the Mining Minds High-Level Context Architecture and the results of its evaluation. Finally, the main conclusions and future steps are presented in Section 6.

## 2. Related Work

A number of surveys have reviewed the use and foundations of ontologies for context modeling. For example, a survey on context-aware systems [16] describes the basic design principles of context-aware architectures and depicts the different context models. Special focus is placed in this survey on the analysis and comparison of several approaches using ontologies. Another review of context modeling and reasoning techniques [17] discusses the requirements for modeling different context information and introduces the concept of high-level context abstractions. This survey describes and compares several ontology-based models of context information. Finally, a more recent survey on context-aware computing for the Internet of Things [18] evaluates 50 projects including the majority of research and commercial solutions proposed in the field of context-aware computing from 2001 to 2011. An extensive evaluation of research prototypes, systems and approaches building on ontology-based modeling and reasoning solutions is presented in this survey.

Many ontologies have been specifically proposed to model and recognize user context. The most well-known context ontologies and ontology-based context frameworks are described in the following. One of the most prominent ontologies for modeling context in pervasive environments is SOUPA (Standard Ontologies for Ubiquitous and Pervasive Applications) [19]. The core of the SOUPA ontology defines generic vocabularies for several domains: person, agent, belief-desire-intention, action, policy, time, space and event. Similarly, CONON (CONtext ONtology) [20] is a noticeable ontology for smart home environments. The CONON upper ontology captures the general features of different context entities: person, activity, computational entity and location. Both SOUPA and CONON ontologies are generic and can be extended to describe the context in the application-specific domain. For example, the Context Broker Architecture (CoBrA) [21] adopts the SOUPA ontology, whereas the SOCAM (Service-oriented Context-Aware Middleware) [22] builds on the CONON ontology. The CoBrA ontology describes places, agents and events in an intelligent meeting room. The ontology proposed in SOCAM models persons, activities, locations and devices for smart home and vehicle environments.

Apart from these early well-known solutions, more recent context ontologies and ontology-based context frameworks have been proposed. The Pervasive Information Visualization Ontology (PIVOn) [23] is composed of four ontologies for the description of intelligent environments: user, device, environment and service. The user model describes the static characteristics of the users, their agenda and their situation, including the user location, the current task and goals. The mIO! ontology [24] models context-related knowledge for the adaptation of applications in mobile environments. This ontology defines concepts like information on location and time, user information and its current or planned activities, as well as devices located in his or her surroundings. The Context Aggregation and REasoning (CARE) middleware [25] performs ontological and statistical reasoning to support the context-aware adaptation of Internet services in a mobile computing environment. The ontology, which models the user context within the CARE middleware, describes the user activities (actions and movements), interests, contacts, calendar items and places. For example, the context business meeting is defined as including any activity performed in a conference room within a company building and having at least two actors, each of which is an employee. Thus, the ontology in the CARE middleware models context based on activities and locations.

Some other works focus on the detection of a specific category of context, mainly activities, sometimes utilizing in their definition other types of contexts, such as locations. ActivO is the ontology used in COSAR [26], an activity recognition system that supports hybrid statistical and ontological reasoning. The ActivO ontology models a set of activities and the context data required to recognize them (the person performing the activity, the location of the activity and the time extent in which the activity takes place). The authors of the ActivO ontology have also proposed a very similar approach, but using OWL2 for modeling and reasoning [13]. Furthermore, some activities involve the interaction with objects. Thus, contextual information about the interaction (time and location) can be used to model and infer the activities. An ontology-based approach is used to model activities for smart homes in [9]. The proposed ontology models activities based on a sequence of user-object interactions and the location of the objects. For instance, the activity *making tea* is composed of the primitives *get cup*, *get tea*, *pour water*, *get milk* and *get sugar*, which take place in the *kitchen* . Composite activities in smart homes are modeled and recognized in [27]. Ontological and temporal knowledge modeling formalisms are combined to describe composite activities, like, for example, *make tea and then wash hands* . The work in [28] describes an ontology-based technique for multilevel activity recognition. The proposed ontology models atomic gestures (actions that cannot be decomposed), manipulative gestures (execution of simple atomic gestures), simple activities (temporal sequences of manipulative gestures) and complex activities (concurrent execution of simple activities). One example of a complex activity could be *clean up*, which is composed of the simple activities *put in dishwasher* and *clean table*. Finally, [29] proposes a fuzzy ontology for the representation of activity and the reasoning on vague, incomplete and uncertain knowledge. The ontology core models three domains: users, environment including locations and actions, activities and behaviors. Actions are atomic events, activities can be a single action or a composed set of actions, and behaviors are a sequence of activities and/or actions. For example, the behavior *coffee break* includes the action *exit office*, the activity *make coffee* or *take coffee* and the action *enter office*.

## 3. Mining Minds Context Ontology

There are multiple reasons supporting the choice of ontologies for context modeling and inference: ontologies surpass non-semantic models for context modeling in terms of flexibility, extensibility, generality, expressiveness and decoupling of the knowledge from the code [30,31,32]. More specifically, ontologies provide implicit semantics that enable the derivation of new information from existing ones, a key characteristic to procure interoperability among different systems. Moreover, the hierarchical structure of ontologies, with subclasses inheriting the properties from their ascendant classes, facilitates its evolvability and maintenance. In fact, new concepts can be easily added to the ontology and related to the existing ones, with multiple existing methods for the automatic validation of conflicts and semantic inconsistencies. Several reasoners are also available and can be used with the defined ontologies. Ontological reasoning can be inherently applied on ontology-based models; thus, simplifying the inference task and avoiding the need for the explicit definition of rules. Finally, using ontologies to infer context is also beneficial from the implementation perspective since no changes are required in the architecture and/or implementation whenever the model is extended; thus, only requiring the adaptation of the ontology itself.

The Mining Minds Context Ontology models context for human behavior identification in order to enable the provision of personalized health and wellness services in Mining Minds [14,15]. Since Dey proposed the first widely-accepted definition of context [33], many different interpretations of context have arisen. In Mining Minds, human context is defined as any information characterizing the physical, mental and social situation of a person that enables the identification of their behavior. Furthermore, human context is here categorized into two different levels of abstraction: low-level context and high-level context. Low-level context is defined as primitive context, i.e., contexts that can be directly identified from user data and do not require any other type of context information to be derived. Specifically, activities, locations and emotions are here considered as the three categories of low-level context. Activities can be normally identified from the body movement; locations can be directly derived from the user position; and emotions can be obtained from the user sentiments or physiological responses. High-level context is the context that requires several contexts of a diverse nature in order to be identified. This means that a high-level context builds on a combination of low-level contexts. Therefore, high-level contexts are more complex and abstract contexts.

**Figure 1:**
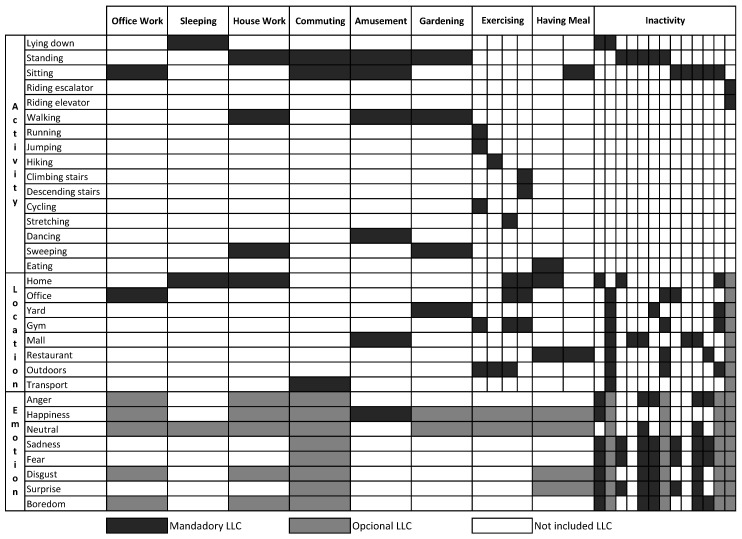
Graphical representation of the combination of low-level contexts that compose the high-level contexts modeled in the Mining Minds Context Ontology.

The Mining Minds Context Ontology aims at comprehensively modeling the most commonplace and widely-used contexts for health and wellness services, such as the ones supported by Mining Minds. These contexts are typically observed for both sedentary and active lifestyles. Specifically, the high-level contexts include daily contexts like *office work*, *sleeping*, *house work*, *commuting*, *amusement*, *gardening*, *exercising*, *having meal* and *inactivity*. The low-level contexts required to compose the description of the high-level context have to be automatically recognizable. Thus, very simple low-level contexts in the domains of activities, locations and emotions are defined. Low-level contexts describing activities include sedentary activities associated with unhealthy habits, mild activities of daily living and some vigorous ones related to sport and fitness practices. Namely, the modeled activities are *lying down*, *standing*, *sitting*, *riding escalator*, *riding elevator*, *walking*, *running*, *jumping*, *hiking*, *climbing stairs*, *descending stairs*, *cycling*, *stretching*, *dancing*, *sweeping* and *eating*. Similarly, the low-level contexts describing the locations comprise the places where the user spends their daily life, i.e., *home*, *office*, *yard*, *gym*, *mall*, *restaurant*, *outdoors* and *transport*. The low-level contexts describing the emotions embrace the most prominent moods or states of mind, which are *anger*, *happiness*, *neutral*, *sadness*, *fear*, *disgust*, *surprise* and *boredom*. The specific combinations of low-level contexts that compose each high-level context are derived from the experience of the Mining Minds behavioral scientists. Figure 1 graphically represents these definitions of high-level context, which are modeled in the Mining Minds Context Ontology. The considered contexts are intended to represent a wide spectrum of situations and actions in a person’s life; however, it must be noted that this list can certainly be extended in view of potential future applications while considering other less recurrent contexts.

In broad strokes, the main novelties of the Mining Minds Context Ontology are a more comprehensive description of context using a two-level model and the incorporation of emotion information to detect some high-level contexts. First, a layered approach is followed in which high-level contexts build on a combination of low-level contexts. Current approaches model context in different dimensions, for example the user is performing an activity, has a location and has a mood. However, in these models, there is no clear link between the different dimensions of context, neither are they used to derive other contexts. Thus, some valuable information for the identification of human behavior is lost when using a one-level model. Second, the emotions enable the definition of new high-level contexts, which can only be identified whenever a specific emotion takes place. This is the case of the high-level context *amusement*, which must imply that the person is happy and having fun. For this context, it is not enough to know that the person is *sitting* in the *mall*, but also that their emotion is *happiness* in order to infer that the context refers to *amusement*. Therefore, in some cases, the activity and the location might not be enough to detect the high-level context, and the emotion enables the identification of more diverse high-level contexts. The Mining Minds Context Ontology is an OWL 2 ontology [34] and is publicly available at [35].

### 3.1. Terminology for the Definition of Context

The Mining Minds Context Ontology defines the concept of user context. The context is associated with a given user and has a start and an end. While a context has necessarily a start referring to the time in which the context initiates, the finalization of the context is not strictly necessary. This is motivated by the fact that the context may be prolonged over time and be still valid at the present time. A given context can refer to either low or high-level context. Low-level contexts represent either activities, locations or emotions, which can further compose a high-level context. In some cases, only one category of the low-level context is enough to determine the high-level context. This is the case of *inactivity*, where a sole sedentary activity like *sitting* defines this context. In some other cases, a specific category of low-level context is essential in order to identify the high-level context. For example, *amusement* can only be detected if the emotion is of type *happiness*. Accordingly, the ontology has been designed to support any combination of low-level contexts to define a specific high-level context. Given the seldom availability of emotion data, the ontology has been designed to procure the identification of some high-level contexts, even in the absence of emotion information. The modeling of this concept of context using the formal ontological description is presented in the following.

**Figure 2:**
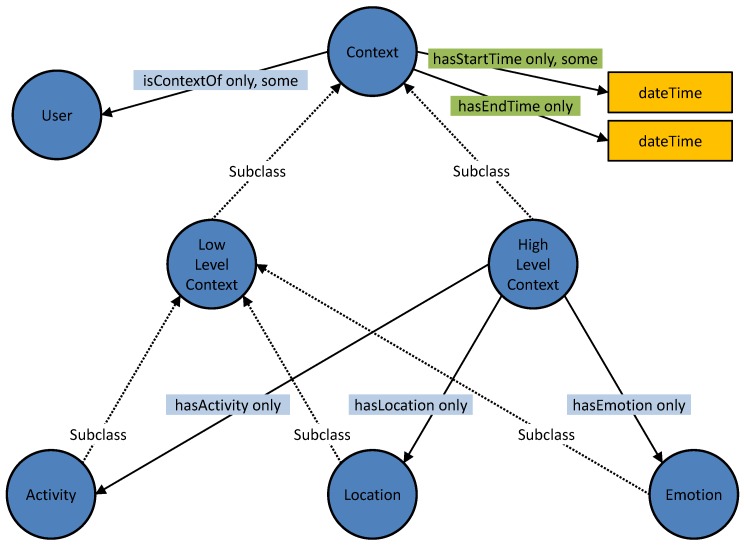
Mining minds context ontology: the class *Context*, its subclasses and the relations among them.

The main concept of the Mining Minds Context Ontology is the class *Context*, which represents the context of a user in an interval of time. Several necessary conditions are described for this class to model the concept of context (Figure 2). The existential and universal restrictions on the object property *isContextOf* ensure that any individual of the class *Context* is linked to an individual of the class *User* representing the user to which the context belongs. The existential and universal restrictions on the functional data property *hasStartTime* state that all of the individuals of the class *Context* must be related along this property to a unique *dateTime* data type of the W3C XML Schema Definition Language (XSD) [36] representing the instant of time in which the context starts. The universal restriction on the functional data property *hasEndTime* indicates that if there is a relationship of an individual of the class *Context* along the property *hasEndTime*, it has to be to a member of the XSD *dateTime* data type representing the end time of the interval in which the context is valid.

**Figure 3:**
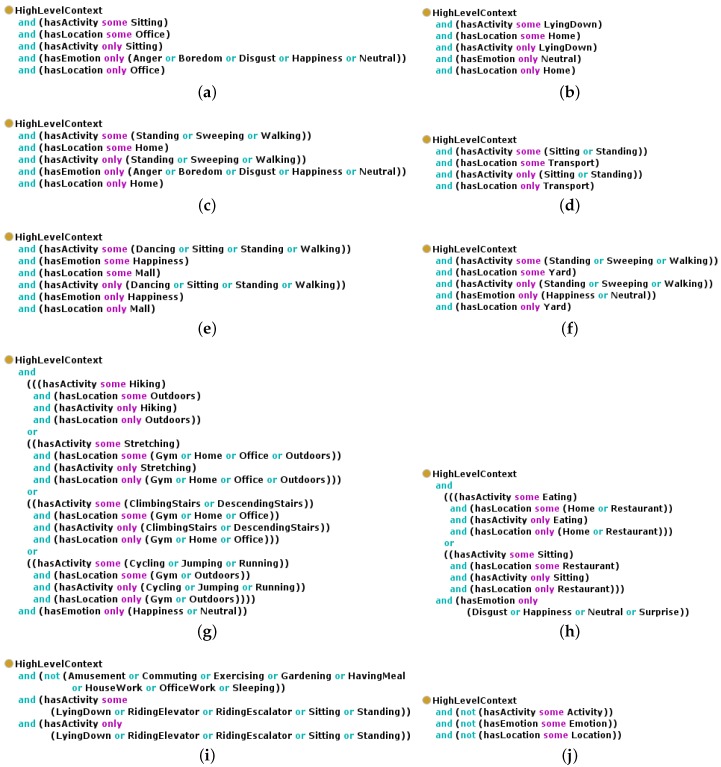
Mining minds context ontology: definition of the ten subclasses of *HighLevelContext*. (**a**) *OfficeWork*; (**b**) *Sleeping*; (**c**) *HouseWork*; (**d**) *Commuting*; (**e**) *Amusement*; (**f**) *Gardening*; (**g**) *Exercising*; (**h**) *HavingMeal*; (**i**) *Inactivity*; (**j**) *NoHLC*.

The class *LowLevelContext* represents the basic categories of low-level contexts via the classes *Activity*, *Location* and *Emotion*. The class *HighLevelContext* models the concept of high-level context. The universal restrictions on the object properties *hasActivity*, *hasLocation* and *hasEmotion* model the relationship between the individuals of the class *HighLevelContext* and the individuals of the different subclasses of *LowLevelContext*, which compose the high level context. The different types of high-level contexts are modeled via ten subclasses of the class *HighLevelContext*. Their equivalent anonymous classes as defined in Protégé [37] are presented in Figure 3.

In order to be a member of the defined class *OfficeWork* (Figure 3a), an individual of the class *HighLevelContext* must have a property of type *hasActivity* which relates to an individual of the class *Sitting*, and this property can only take as a value an individual of the class *Sitting*. Moreover, the individual of the class *HighLevelContext* must also have a property of type *hasLocation*, which relates to an individual of the class *Office* and only to an individual of the class *Office*. Finally, in case the individual of the class *HighLevelContext* has a property of type *hasEmotion*, this property must relate to an individual of the class *Anger*, the class *Boredom*, the class *Disgust*, the class *Happiness* or the class *Neutral*. This universal restriction does not specify that the relationship along the property *hasEmotion* must exist, but if it exists, it must link to the specified class members.

### 3.2. Instances of Context

**Figure 4:**
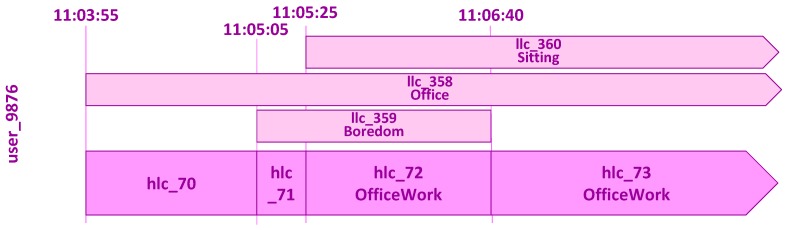
Exemplary scenario representing low-level contexts and high-level contexts.

An illustrative scenario is presented here to showcase the representation of instances of low-level contexts and high-level contexts in the Mining Minds Context Ontology (Figure 4). Let us imagine that it is 10 November 2015, and the user with identifier 9876 enters at 11:03:55 the office building of her or his working place. This event is detected by a location detector, a positioning system that interprets the coordinates of the user as the location of her or his office. Therefore, the low-level context of category location is identified as being of type *office* at 11:03:55. She or he starts talking on the phone, and a system capable of recognizing emotions detects from the tone of her or his voice that the user is bored. Thus, the low-level context of category emotion is identified as being of type *boredom* at 11:05:05. The phone call finalizes at 11:06:40, and then, no emotion is detected anymore. Meanwhile, at 11:05:25, the user sits down at her or his workplace. This event is detected by an activity recognizer that continuously measures her or his body motion. The low-level context of category activity is identified as being of type *sitting* at 11:05:25. It should be noted that every change in any of the low-level contexts may potentially lead to a new high-level context. For example, at 11:05:05, the combination of the activity *sitting*, the location *office* and the emotion *boredom* creates a high-level context that is classified as *office work*. At 11:06:40, when the emotion is no longer available, but the activity remains as *sitting* and the location as *office*, the high-level context for this user continues being identified as *office work*. Some combinations of low-level contexts do not constitute a known class of high-level context, based on the defined ontology. This is the case of the two high-level contexts at the beginning of this scenario. Namely, only location or the combination of the location *office* and the emotion *boredom* turn out to be not enough to identify a more abstract high-level context. Each context has associated a name, which serves as a unique identifier. These names are automatically created by the system whenever a new context is detected and are composed of the prefix *“llc_”* or *“hlc_”* and a sequential unique number. For the sake of simplicity, in this example, up to three digits are considered; however, large numbers are normally used by the system to procure unique identifiers. Furthermore, in order to make the example more understandable, for the low-level contexts, the membership of the instance to its name has been appended. For example, the context representing the activity *sitting* is named *llc_360_sitting*.

The terminology described in Section 3.1 is utilized at this point to generate the instances of context resulting from this scenario. The instances of low-level context are directly created from the information provided by the activity recognizer, location detector or emotion recognizer. In Section 3.2.1, the generation of the low-level contexts is presented. High-level contexts can be created from the information of the low-level contexts which are part of it and which triggered its occurrence. In Section 3.2.2, the generation of the high-level contexts is introduced. High-level contexts can also be classified, i.e., the membership of the high-level context or the class to which a high-level context belongs can be determined. In Section 3.2.3, the inference of the membership of the high-level contexts is described. Since the process of inferring the membership of a high-level context is also called classification, the high-level contexts for which their membership has been inferred are hereafter called *classified* high-level contexts. Conversely, the high-level contexts for which their membership has not been inferred are hereafter called *unclassified* high-level contexts. Finally, it is possible that the classification of an unclassified high-level context does not result in any inferred statement. In other words, the high-level context does not belong to any of the classes of high-level context defined in the terminology. In this case, the high-level context, which has been intended to be classified, but does not belong to any known class, is called *unidentified* high-level context.

#### 3.2.1. Instances of Low-Level Context

**Figure 5:**
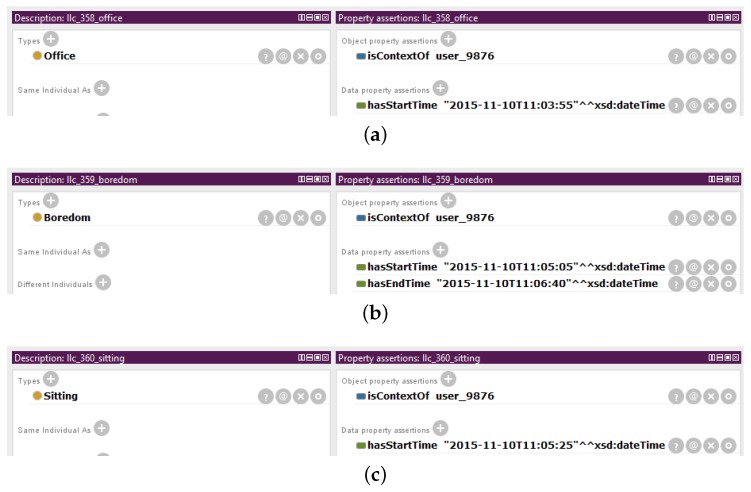
Representation of the instances of low-level context for the exemplary scenario by using the Mining Minds Context Ontology in Protégé. (**a**) *llc_358_office* is a member of the class *Office*; (**b**) *llc_359_boredom* is a member of the class *Boredom*; and (**c**) *llc_360_sitting* is a member of the class *Sitting*.

The low-level contexts are modeled as members of the subclasses of *LowLevelContext*: *Activity*, *Location* and *Emotion*. Figure 5 shows how the low-level contexts for the presented scenario are described in Protégé. *llc_358_office*, *llc_359_boredom* and *llc_360_sitting* are members of the classes *Office*, *Boredom* and *Sitting*, respectively. These instances model the low-level context of the user with identifier 9876. Thus, *llc_358_office*, *llc_359_boredom* and *llc_360_sitting* are related along the property *isContextOf* to the individual *user_9876*, which is a member of the class *User*. All of the individuals representing the low-level contexts have a relationship along the property *hasStartTime* to a value in the form of XSD *dateTime*, which represents the start time of the interval in which the low-level context is valid. For example, for the individual *llc_359_boredom*, the property *hasStartTime* links to the value *“2015-11-10T11:05:05”ˆˆdateTime*, which indicates that this context started at 11:05:05 on 10 November 2015. Moreover, for this very individual, the property *hasEndTime* relates to the value *“2015-11-10T11:06:40”ˆˆdateTime*, which means that this low-level context only occurred until 11:06:40 on 10 November 2015. Therefore, the individual *llc_359_boredom* models a low-level context of the type *boredom* for the user with identifier 9876 and which was valid in the period of time comprising from 11:05:05 to 11:06:40 on 10 November 2015.

#### 3.2.2. Instances of Unclassified High-Level Context

The unclassified high-level contexts are modeled as members of the class *HighLevelContext* for which their properties and types are stated. Property assertions are used to define the low-level contexts that compose the unclassified high-level context. The properties *hasActivity*, *hasLocation* and *hasEmotion* relate to the individuals of the subclasses of the classes *Activity*, *Location* and *Emotion*, respectively. Reasoning in OWL is based on the Open World Assumption (OWA), which means that it cannot be assumed that something does not exist unless it is explicitly stated that it does not exist. Therefore, type assertions are used as closure axioms to indicate that an unclassified high-level context is composed of a unique and finite set of low-level contexts. Specifically, for each of the low-level context components of the high-level context, it is stated the type equivalent to the anonymous class represented by the universal restriction on the property *hasActivity*, *hasLocation* or *hasEmotion* where the value of the filler is the collection comprising only the low-level context. Furthermore, type assertions are also used as closure axioms to indicate that there is no low-level context of a specific category being part of the unclassified high-level context. In this case, for each of the categories of low-level context absent on the unclassified high-level context, it is stated that the type equivalent to the anonymous class is the negation class of the existential restriction on the property *hasActivity*, *hasLocation* or *hasEmotion* where the filler is the class representing the category of low-level context, *Activity*, *Location* or *Emotion*, respectively.

**Figure 6:**
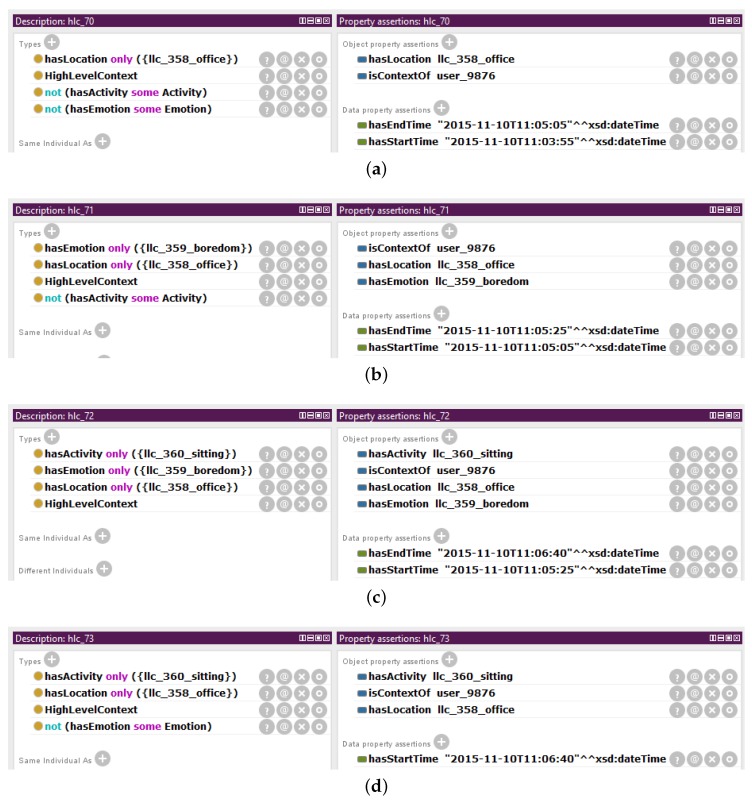
Representation of the instances of unclassified high-level context for the exemplary scenario by using the Mining Minds Context Ontology in Protégé. (**a**) *hlc_70*; (**b**) *hlc_71*; (**c**) *hlc_72*; and (**d**) *hlc_73* are composed of some of the low-level contexts *llc_358_office* (member of the class *Office*), *llc_359_boredom* (member of the class *Boredom*) and *llc_360_sitting* (member of the class *Sitting*).

Figure 6 shows how the unclassified high-level contexts for the presented scenario are described in Protégé. *hlc_70*, *hlc_71*, *hlc_72* and *hlc_73* are members of the class *HighLevelContext*. Similarly as for the low-level contexts, the individuals representing the unclassified high-level contexts have relationships along the properties *isContextOf*, *hasStartTime* and *hasEndTime*. For the individual *hlc_72*, the property *hasActivity* relates to the individual *llc_360_sitting*, the property *hasLocation* to the individual *llc_358_office* and the property *hasEmotion* to the individual *llc_359_boredom*. Due to the OWA, *hlc_72* has been asserted as the type *hasActivity only ({llc_360_sitting})*, the type *hasLocation only ({llc_358_office})* and the type *hasEmotion only ({llc_359_boredom})*. These statements indicate that the individual *hlc_72* only has a *hasActivity* relationship to *llc_360_sitting*, a *hasLocation* relationship to *llc_358_office* and a *hasEmotion* relationship to *llc_359_boredom*. The individual *hlc_73* is composed of the same activity and location as *hlc_72*; however, no emotion is part of this unclassified high-level context. Therefore, *hlc_73* has been asserted as the type *not (hasEmotion some Emotion)*. This statement indicates that the individual *hlc_73* does not have any property of type *hasEmotion* linking to an individual of the class *Emotion*, i.e., this unclassified high-level context does not contain any emotion.

#### 3.2.3. Instances of Classified High-Level Context

**Figure 7:**
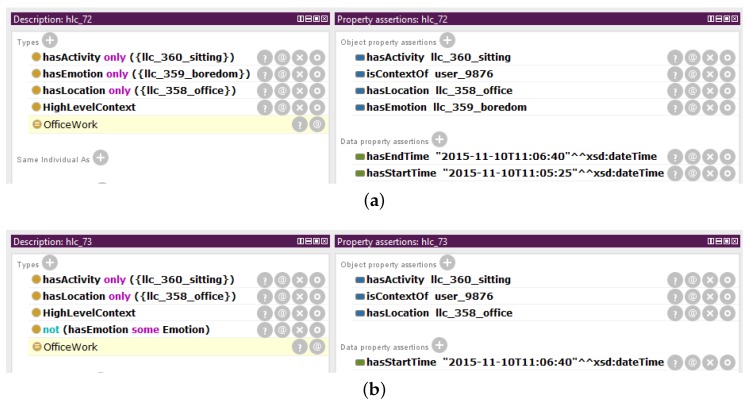
Representation of the instances of classified high-level context for the exemplary scenario by using the Mining Minds Context Ontology in Protégé. (**a**) *hlc_72*; and (**b**) *hlc_73*, which are both inferred to be members of the class *OfficeWork*, are composed of some of the low-level contexts *llc_358_office* (member of the class *Office*), *llc_359_boredom* (member of the class *Boredom*) and *llc_360_sitting* (member of the class *Sitting*).

The classified high-level contexts are obtained using a reasoner that infers the membership of the unclassified high-level contexts. Thus, a classified high-level context is an individual of the class *HighLevelContext*, which is determined to be also a member of one of the ten subclasses of *HighLevelContext*: *OfficeWork*, *Sleeping*, *HouseWork*, *Commuting*, *Amusement*, *Gardening*, *Exercising*, *HavingMeal*, *Inactivity* or *NoHLC*. Figure 7 shows the classified high-level contexts for the working scenario and that have been inferred in Protégé using the Pellet reasoner [38]. The individuals *hlc_70* and *hlc_71* are not presented in the figure since they do not belong to any known class of high-level context, i.e., they are unidentified high-level contexts.

The individual *hlc_72* is inferred by the reasoner to belong to the class *OfficeWork* (Figure 7a). Since this individual of the class *HighLevelContext* complies with the definition of the class *OfficeWork*, it is classified as being a member of this class. *hlc_72* fulfills the existential and universal restrictions on the property *hasActivity*, which state that a member of the class *OfficeWork* must have some *hasActivity* relationship to an individual of the class *Sitting* and only to a member of this class. These restrictions are met since the property *hasActivity* only links the individual *hlc_72* to the individual *llc_360_sitting*, which is a member of the class *Sitting*. Similarly, *hlc_72* also fulfills the existential and universal restrictions on the property *hasLocation*. Furthermore, *hlc_72* fulfills the universal restriction on the property *hasEmotion*, which states that in the case a member of the class *OfficeWork* has a *hasEmotion* relationship, it has to link to only an individual of the class *Boredom*. In fact, *hlc_72* is only related along the property *hasActivity* to the individual *llc_359_boredom*, which is a member of the class *Boredom*.

The individual *hlc_73* is also classified by the reasoner as being a member of the class *OfficeWork* (Figure 7b). Similar to the classified high-level context *hlc_72*, the individual *hlc_73* also complies with the existential and universal restrictions on the properties *hasActivity* and *hasLocation*. However, the property *hasEmotion* about the individual *hlc_73* is not asserted. The universal restriction on the property *hasEmotion* does not state that the relationship must exist. In fact, it may not exist at all and the restriction still be fulfilled, as is the case for *hlc_73*. Thus, the individual *hlc_73* can be inferred as being a member of the class *OfficeWork*. The classification as members of the class *OfficeWork* of the two individuals of the class *HighLevelContext*, *hlc_72* and *hlc_73*, one with a *hasEmotion* relationship and another without it, proves the flexibility of the Mining Minds Context Ontology, which enables the identification of high-level contexts, even if one of the pieces of low-level information is missing. This is considered to be helpful in real-life scenarios where emotion recognition systems are not always available or may generate detection events in a less regular basis than activity recognizers or location detectors.

## 4. Mining Minds High-Level Context Architecture

**Figure 8:**
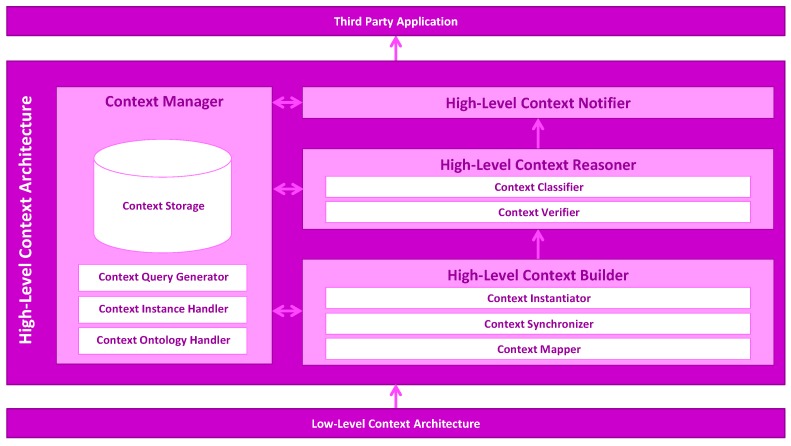
Mining Minds High-Level Context Architecture.

The Mining Minds High-Level Context Architecture (HLCA) is the system architecture devised for the identification of the user context in Mining Minds. Conversely to most similar approaches, the HLCA supports the instance-based identification of context. The HLCA infers abstract context representations based on categories, such as physical activities, emotional states and locations. These categories, which are derived from the wide-spectrum of multimodal data obtained from the user interaction with the real- and cyber-world, are intelligently combined and processed at the HLCA in order to determine and track the user context. The inferred user context can be utilized by the Mining Minds entities and any other third party to enable the provision of personalized health and wellness services. The HLCA relies on the Mining Minds Context Ontology (Section 3) and applies ontological inference to identify the user context. Furthermore, the ontology is utilized in the representation of the context shared between the components of the HLCA. The HLCA consists of four main components (Figure 8): High-Level Context Builder (Section 4.1), High-Level Context Reasoner (Section 4.2), High-Level Context Notifier (Section 4.3) and Context Manager (Section 4.4).

In a nutshell, the operation of the HLCA is as follows. The High-Level Context Builder receives unstructured low-level information, namely activities, emotions and locations, yielded by the Low-Level Context Architecture, an independent Mining Minds entity that is in charge of identifying the user low-level context using data driven-approaches. Then, based on the received low-level context information, the High-Level Context Builder generates the ontological concepts representing the user context. The Context Mapper is in charge of interpreting the received low-level information and transforming it into the corresponding ontological concepts. The Context Synchronizer seeks concurrent low-level contexts, identifying other user contexts valid at the same moment in time. The Context Instantiator generates a new instance of an unclassified high-level context linking to the low-level contexts that compose it. The High-Level Context Reasoner receives the unclassified high-level context for its verification and classification. The Context Verifier checks the semantic and syntactic consistency of the unclassified high-level context. The Context Classifier identifies the membership of the unclassified high-level by applying ontological inference. The High-Level Context Notifier makes available the newly-classified high-level context to any third party application that registered for this type of information. During the context identification process, several components interact with the Context Manager, which provides the persistence of the Mining Minds Context Ontology, as well as supports the easy access to low-level context and high-level context information.

In the following, the different components of the HLCA are described in detail. For the sake of understanding, an example from the scenario presented in Section 3.2 is here considered to illustrate the operation of each component of the HLCA. Namely, the inference of a new high-level context at 11:05:25 on 10 November 2015 is considered. At that moment, a new low-level context of the category *sitting* for the user with identifier 9876 is detected by the Low-Level Context Architecture. This event triggers the operation of the HLCA, which after the processing identifies a new high-level context of type *office work* and serves it to the registered third party applications.

### 4.1. High-Level Context Builder

The High-Level Context Builder receives the low-level information, i.e., activities, emotions and locations, and generates the ontological concepts representing an unclassified high-level context associated with that information. The High-Level Context Builder has three subcomponents: the Context Mapper, the Context Synchronizer and the Context Instantiator.

#### 4.1.1. Context Mapper

The Context Mapper interprets the received low-level information and transforms it into the corresponding ontological concepts. Specifically, it maps the labels plus metadata into ontological instances of low-level context. Whenever the Context Mapper gets a new label, it creates an instance of the subclass of the class *LowLevelContext* which represents the corresponding activity, location or emotion (as described in Section 3.2.1). The property *hasStartTime* is stated to relate this instance to the time in which the low-level context started and which is part of the received metadata. Furthermore, the user to which the context belongs is related along the property *isContextOf*. Once the low-level context instance has been created, it is stored in the Context Manager for its persistence (see Section 4.4.3) and it is notified to the Context Synchronizer.

For the working example, the Context Mapper receives at run-time the activity label “sitting” and several metadata, i.e., the identifier of the user “9876” and the time in which the context starts “2015-11-10T11:05:25”. The Context Mapper generates an instance of low-level context and then asserts the properties about it. The instance *llc_360_sitting* of the class *Sitting* presented in Figure 5c is created. This instance has a *isContextOf* relationship to the individual *user_9876* and a *hasStartTime* relationship to the value *“2015-11-10T11:05:25”ˆˆdateTime*.

#### 4.1.2. Context Synchronizer

The Context Synchronizer searches for concurrent low-level contexts, whenever the Context Mapper has notified a newly detected low-level context instance. A change in the low-level context implies a new high-level context, comprising the new low-level context and the other low-level contexts still valid at the start of the new low-level context. The Context Synchronizer needs to determine the other low-level contexts of a given user which are valid a the start time of the new low-level context instance created by the Context Mapper. Therefore, one of the most important roles of the Context Synchronizer is to align concurrent low-level contexts of the same user which might have been received in an unordered manner due to the diverse delays introduced by the different components of the Low-Level Context Architecture. In order to search for the concurrent low-level contexts, the Context Synchronizer requests information stored in the Context Manager and accesses it through the Context Instance Handler (see Section 4.4.3). Once the Context Synchronizer has determined the low-level contexts concurrent to the one that triggered the process, the Context Instantiator is invoked.

In the considered example, when the Context Synchronizer is notified by the Context Mapper about the identification of the new low-level context represented by the instance *llc_360_sitting*, it searches for concurrent low-level contexts by querying the information stored in the Context Manager. The instances *llc_358_office* and *llc_359_boredom*, presented in Figure 5a,b, are found to be concurrent to the low-level context *llc_360_sitting*. These two low-level contexts belong to the same user, i.e., user with identifier 9876, and they are still valid at 11:05:25 on 10 November 2015, when the new low-level context *sitting* starts.

#### 4.1.3. Context Instantiator

The Context Instantiator creates a new instance of an unclassified high-level context linking to the constituent low-level contexts. Whenever the Context Synchronizer detects a set of low-level contexts which are concurrent to a newly detected one, the Context Instantiator creates a new instance of an unclassified high-level context containing these low-level contexts (as described in Section 3.2.2). Therefore, an instance of the class *HighLevelContext* is created and the different low-level contexts which compose the high-level context are related to it along the properties *hasActivity*, *hasLocation*, and *hasEmotion*. Moreover, the closure axioms are established via type assertions on these properties. In case there is a low-level context of a particular type, the Context Instantiator generates the axiom stating that the property can only link to that given low-level context. Otherwise, if no low-level context has been determined for one of the categories-activities, locations or emotions-, the Context Instantiator creates the axiom stating that there is no low-level context of that category. Furthermore, the Context Instantiator establishes a *hasStartTime* relationship to the time in which the high-level context change happened, i.e., the time in which the newly detected low-level context started and which triggered the creation of the new unclassified high-level context. Moreover, the user to which the high-level context belongs is related along the property *isContextOf*. The identifier of the user to which the high-level context belongs is the same than the one associated to the low-level contexts which compose the high-level context. Once the Context Instantiator has created the instance of an unclassified high-level context, this is served to the High-Level Context Reasoner (see Section 4.2) for its verification and classification.

For the working example, the Context Instantiator receives from the Context Synchronizer the newly detected low-level context represented by the instance *llc_360_sitting* and the concurrent low-level contexts *llc_358_office* and *llc_359_boredom*. The Context Instantiator creates the instance *hlc_72* of the class *HighLevelContext* (see Figure 6c) and links it to the low-level contexts which compose it. Therefore, the properties *hasActivity*, *hasLocation*, and *hasEmotion* relate, respectively, to the instances *llc_360_sitting*, *llc_358_office*, and *llc_359_boredom*. The closure axiom *hasActivity only ({llc_360_sitting})* indicates that the individual *hlc_72* only has a *hasActivity* relationship to the individual *llc_360_sitting*. Similarly, the other two closure axioms, *hasLocation only ({llc_358_office})* and *hasEmotion only ({llc_359_boredom})*, state the uniqueness of the relationships. The Context Instantiator also specifies that the instance *hlc_72* has a *isContextOf* relationship to the individual *user_9876* which is the owner of the different low-level contexts composing the high-level context. Finally, the Context Instantiator creates a relationship along the property *hasStartTime* to the moment in which the change in the low-level context triggered the identification of the new high-level context. The start time of the high-level context *hlc_72* is the start time of the low-level context *llc_360_sitting*. Thus, for the instance *hlc_72* the property *hasStartTime* links to the value *“2015-11-10T11:05:25”ˆˆdateTime*.

### 4.2. High-Level Context Reasoner

The High-Level Context Reasoner performs a consistency check on the unclassified high-level context instance created by the High-Level Context Builder (see Section 4.1). In case the instance is valid, the High-Level Context Reasoner identifies the context type to which the high-level context belongs, i.e., it classifies the high-level context instance. In order to perform these tasks, the High-Level Context Reasoner applies ontological inference supported by the formal description of context in the Mining Minds Context Ontology (see Section 3.1). The High-Level Context Reasoner comprises two subcomponents: the Context Verifier and the Context Classifier.

#### 4.2.1. Context Verifier

The Context Verifier checks the semantic and syntactic consistency of the unclassified high-level context provided by the High-Level Context Builder. Therefore, the instance of unclassified high-level context is validated and verified versus the Mining Minds Context Ontology, which is stored in the Context Manager and can be accessed through the Context Ontology Handler (see Section 4.4.2). During the consistency check, non-logical or malformed high-level contexts can be detected. For example, the high-level contexts which do not contain the necessary property *hasStartTime* or the ones composed from multiple different instances of low-level contexts of the same type. Once the Context Verifier has ensured that the unclassified high-level context is valid, this instance is provided to the Context Classifier for further processing.

In the described example, the Context Verifier receives from the Context Instantiator the newly created high-level context *hlc_72*. This instance is checked for its semantic and syntactic consistency, it is considered to be valid, and it is then served to the Context Classifier.

#### 4.2.2. Context Classifier

The Context Classifier identifies the type of high-level context to which the unclassified high-level context belongs; thus, converting the unclassified instance into a classified high-level context. The classification of the unclassified high-level context instance into one of the defined high-level context classes is based on the inference functionalities provided by the Mining Minds Context Ontology. Specifically, one of the key features of this ontology is that it can be processed by a reasoner which can automatically perform the classification process. This means that the unclassified high-level context instance is compared versus the definitions of the different high-level context classes to determine whether it complies with the conditions that define the class. In case it complies, the instance is inferred to belong to that class. The classification process is triggered every time the Context Classifier receives a new valid instance of high-level context from the Context Verifier. After the membership of the unclassified high-level context instance has been determined, the Context Classifier adds to the unclassified high-level context instance the axiom stating that this instance belongs to a specific type of high-level context. Therefore, the instance of the class *HighLevelContext* which models the classified high-level context is related along the property *rdf:type* to the subclass of the class *HighLevelContext* representing the high-level context of which the instance is a member. It is possible that the unclassified high-level context does not belong to any of the known classes described in the Mining Minds Context Ontology. This means that no membership is inferred and the unclassified high-level context is considered to belong to an unidentified type of high-level context. In this case, the classified high-level context has the same exact representation than the corresponding unclassified high-level context. Finally, the Context Classifier serves the classified high-level context to the High-Level Context Notifier (see Section 4.3).

For the working example, the Context Classifier receives from the Context Verifier the high-level context *hlc_72*. The Context Classifier applies the classification method to this unclassified high-level context in order to determine its membership. The individual *hlc_72* is inferred to belong to the class *OfficeWork* since it complies with the definition of the class *OfficeWork* (as described in Section 3.2.3). Therefore, the Context Classifier creates the axiom *hlc_72 rdf:type OfficeWork* which indicates that the individual *hlc_72* is a member of the class *OfficeWork*. The classified high-level context instance *hlc_72* is provided to the High-Level Context Notifier for its notification.

### 4.3. High-Level Context Notifier

The High-Level Context Notifier makes available to third party applications the newly identified high-level contexts. The High-Level Context Notifier receives from the High-Level Context Reasoner a classified high-level context instance and notifies the subscribed third parties about the detection of a new high-level context. This notification is only conducted if the new instance belongs to a high-level context type different than the previous one. Only changes in the high-level context type are notified, this means that differences in the low-level context composition which do not imply a change on the type of high-level context are not communicated to the third parties. Furthermore, the High-Level Context Notifier stores the new high-level context into the Context Manager for its persistence via the Context Instance Handler (see Section 4.4.3) and gets as an answer from this component the previous valid high-level context.

For the described example, the High-Level Context Notifier receives from the High-Level Context Reasoner the high-level context *hlc_72* which has been classified as *OfficeWork*. The High-Level Context Notifier contacts the Context Instance Handler for the persistence of the instance *hlc_72* into the Context Storage. Moreover, the High-Level Context Notifier receives from the Context Instance Handler the previous valid instance of high-level context *hlc_71*. The High-Level Context Notifier compares the membership of *hlc_72* to the membership of the previous valid high-level context *hlc_71*. The High-Level Context Notifier determines that there has been a change in the type of high-level context, the previous instance *hlc_71* was unidentified and the new instance *hlc_72* is *office work*. Therefore, the third parties are notified about the change in the high-level context modeled as the instance *hlc_72*.

### 4.4. Context Manager

The Context Manager persists the Mining Minds Context Ontology, including the terminology for the definition of context and the instances of context. Furthermore, this component eases the interactions with the persisted context information by facilitating the exchanges with the storage infrastructure. The Context Manager has four subcomponents: the Context Storage, the Context Ontology Handler, the Context Instance Handler and the Context Query Generator.

#### 4.4.1. Context Storage

The Context Storage is a database which provides persistence for the storage of the Mining Minds Context Ontology, including both the context definition terminology and the context instances. Since the context is modeled via an ontology and the context instances are represented as ontological instances, this storage is devised to be a database of the type triple store. Moreover, the Context Storage also provides read and write functionalities for the Mining Minds Context Ontology. However, this storage cannot be directly accessed and all the interactions are handled through the Context Ontology Handler and the Context Instance Handler.

#### 4.4.2. Context Ontology Handler

The Context Ontology Handler provides the management functionalities to interact with the Mining Minds Context Ontology terminology stored in the Context Storage. This component enables loading the context ontology to the Context Storage at the system start time. The Context Ontology Handler also supports the retrieval of the context ontology which is stored in the Context Storage, so that the rest of components of the HLCA have access to the latest version of the ontological terminology. Furthermore, the Context Ontology Handler enables the extension at runtime of the context ontology. The extensibility is required to evolve the context ontology, therefore, including new types of low-level contexts and new definitions for the high-level contexts. Every time the ontology is updated, the rest of components of the HLCA making direct use of the context ontology are notified to obtain an updated version of the terminology.

#### 4.4.3. Context Instance Handler

The Context Instance Handler deals with the retrieval and storage of context information in the Context Storage. The Context Instance Handler offers three different functionalities: storage of a newly mapped low-level context, retrieval of concurrent low-level contexts, and storage of a newly inferred high-level context while retrieving the previous valid high-level context. The Context Instance Handler poses to the Context Storage the SPARQL queries [39] created by the Context Query Generator in order to retrieve the persisted context information. Specifically, the logic of the Context Instance Handler for the storage of a newly inferred high-level context is as follows. The identification of a new high-level context implies that the previous context for the given user is not valid anymore. Therefore, the storage process includes the finalization of the previous valid high-level context instance. This operation entails to set the value of the end time of the previous valid high-level context stored in the Context Storage. In order to find the previous valid high-level context, the Context Instance Handler needs to pose the appropriate SPARQL queries to the Context Storage. The Context Query Generator is invoked to create the queries for the previous valid high-level context based on the newly inferred high-level context instance (see Section 4.4.4). Furthermore, it must be noted that a an earlier new high-level context could be inferred after the classification of a posterior one. This scenario is not very common but could happen due to the different delays in the data-driven recognition process for the low-level contexts. If this situation occurs, the newly inferred high-level context is only valid until the start time of the posterior high-level context already stored in the Context Storage. Therefore, the storage process also includes the finalization of the newly inferred high-level context instance.

In the considered example, the High-Level Context Notifier interacts with the Context Instance Handler to persist the newly classified high-level context instance *hlc_72* and to retrieve the previously valid instance of high-level context. Therefore, the Context Instance Handler stores the instance *hlc_72* into the Context Storage. Moreover, the Context Instance Handler retrieves from the Context Storage the previously valid instance of high-level context. The previous high-level context is here an individual of the class *HighLevelContext* modeling the context of the user represented by the individual *user_9876* and which is valid at at 11:05:25 on 10 November 2015. In order to retrieve the previous high-level context for the instance *hlc_72*, the Context Instance Handler invokes the Context Query Generator which creates the SPARQL queries presented in Listing 1. This query is posed to the Context Storage which returns as the matching result the high-level context *hlc_71*. Then, the Context Instance Handler finalizes the previous high-level context instance *hlc_71*. This means that the individual *hlc_71* is related along the property *hasEndTime* to the value *“2015-11-10T11:05:25”ˆˆdateTime*, which is the value for the property *hasStartTime* of the newly identified high-level context *hlc_72*. In this exemplary scenario, it is assumed that there are no delays in the recognition of the low-level contexts and therefore, there are no high-level contexts posterior to *hlc_72* which had already been detected.

#### 4.4.4. Context Query Generator

The Context Query Generator is the component which generates the SPARQL queries [39] required by the Context Instance Handler in order to find the matching context instances stored in the Context Storage. The SPARQL queries are automatically created based on some information derived from the context instance that the Context Instance Handler provides to the Context Query Generator. The Context Query Generator is capable of generating several different SPARQL queries depending on the expected outcome required for each specific use case scenario. The Context Query Generator creates SPARQL queries for the identification of a low-level context still valid at the start time of a newly recognized low-level context, which belongs to the very user and which is of the same context category. The Context Query Generator also creates SPARQL queries for the identification of the start time of the next posterior low-level context which belongs to the actual user and which is of the same context category. The Context Query Generator can also create SPARQL queries for the identification of low-level contexts of a given user which are concurrent at the start time of a newly recognized low-level context instance. In addition, the Context Query Generator creates SPARQL queries for the identification of a high-level context which is still valid at the start time of a new high-level context and which belongs to the same user. Finally, the Context Query Generator creates SPARQL queries for the identification of the start time of the next posterior high-level context belonging to the same user.

The logic for the creation of SPARQL queries for the identification of a high-level context which is still valid at the start time of a new high-level context and which belongs to the same user is the following. There are two cases in which the previous high-level context is still valid, either it does not have an end time or its end time is posterior to the start time of the new high-level context. In the first case, the SPARQL needs to match a high-level context for the same user which has a start time previous to the start time of the new high-level context but does not have an end time. In the second case, the SPARQL needs to match a high-level context for the same user which has a start time previous to the start time of the new high-level context and an end time posterior to the start of the new high-level context.

The specific SPARQL query to request the previous high-level context for the instance *hlc_72* is presented in Listing 1. In the considered example, the previous high-level context for *hlc_72* is an individual of the class *HighLevelContext* which belongs to the user represented by the individual *user_9876* and which is valid at 11:05:25 on 10 November 2015. Therefore, the matching individual has to be a member of the class *HighLevelContext*, must have a *isContextOf* relationship to the individual *user_9876*, must have a *hasStartTime* relationship to a value less than or equal to *“2015-11-10T11:05:25”ˆˆdateTime*, and must not have any *hasEndTime* relationship.

Listing 1 **SPARQL query to request the previous high-level context for the instance *hlc_72*.**

**SELECT** ?hlc
**WHERE**
  { ?hlc rdf:type HighLevelContext ;
      isContextOf user_9876 ;
      hasStartTime ?starttime .
   **FILTER NOT EXISTS** ?hlc hasEndTime ?endtime .
   **FILTER** ( ?starttime <= “2015-11-10T11:05:25”ˆˆxsd:dateTime )
}

## 5. Evaluation

This section analyzes both the robustness and performance of the Mining Minds High-Level Context Ontology and Architecture. Section 5.1 explores the tolerance offered by the Mining Minds Context Ontology for the inference of high-level contexts under the presence of low-level context errors. Section 5.2 studies the performance of the Mining Minds High-Level Context Architecture with respect to processing time and management of context instances.

### 5.1. Robustness of the Mining Minds Context Ontology

The proposed Mining Minds Context Ontology has been evaluated to determine how robust the identification of high-level contexts can be in the event of having erroneously detected low-level contexts. In other words, this evaluation aims at measuring the level of resilience of the high-level context level against errors originated at the low-level context level. Pellet (v2.3.2) [38], an open source OWL DL reasoner for Java has been used in the evaluation test program. First, a set of 1800 instances representing all the possible combinations of low-level contexts, i.e., activities, locations and emotions, have been generated. Then, the instances have been posed to the reasoner and the corresponding high-level contexts have been inferred. The resulting array of high-level contexts represents the ground-truth for this evaluation. Subsequently, various scenarios with increasing levels of error in the low-level contexts have been defined. Namely, 5, 10, 20 and 50 per cent of errors have been respectively introduced in the 1800 instances as to emulate potentially erroneous low-level contexts. For example, in the case of having a 10% of affected instances a total of 180 randomly selected instances are deliberately modified. The error has been introduced by replacing the supposedly affected low-level context with a new value randomly selected from the remaining contexts in the affected category (activity, location or emotion). Thus for example, if the original instance of high-level context is composed of the contexts *sitting*, *office* and *boredom*, and the affected context is the activity, the newly generated instance could contain the contexts *running*, *office* and *boredom*. Moreover, in order to evaluate the prominence of each specific context category or combination thereof, the analysis has been formulated for all the combinations of low-level categories, i.e., introducing errors in solely the activity, location, emotion, or combination of activity and location, activity and emotion, location and emotion, and all activity, location and emotion. The instances resulting from all these scenarios have been posed to the reasoner and the resulting high-level contexts have been compared against the ground truth to determine the accuracy of the model. Each of the experiments has been repeated one hundred times in order to ensure the statistical robustness. The average and standard deviation accuracy is presented in Table 1 for each corresponding study.

From an overall analysis of the obtained results it can be concluded that the impact of the error introduced in the low-level context is generally lower at the high-level context. For example, in the case of introducing a 5% error, the accuracy drops approximately no more than 0.4% at best and 3.5% in the worst case scenario. Similarly, for the 10%, 20% and 50% error cases the minimum and maximum accuracy drops are below the corresponding level of error. Experiencing a lesser impact is generally due to the fact that not all the misrecognitions at the low-level context lead to an inference error at the high-level context. For example, if the activity *running* is recognized instead as *climbing stairs*, and provided the rest of low-level contexts to be *gym* for the location and *neutral* for the emotion, the inferred high-level context remains to be *exercising*. Similar examples in which the error in the low-level context is not propagated to the high-level context can be found for the case of erroneous locations and emotions. It can also be observed that the activity is the most prevalent category in terms of error impact, which is certainly as a consequence of the importance given to the activity context in the definition of high-level contexts. Conversely, the location and especially the emotion tend to show a lower effect on the high-level context. In fact the definition of some high-level contexts allows for a good level of resilience against errors in the locations and the emotions. This is the case of the high-level context *inactivity*, which is determined from a sole sedentary activity, like *lying down*, and nearly any location and emotional state. Therefore, even if an the location is erroneously detected, the inferred high-level context would result in *inactivity*. The only exception to this case would happen if the location is misrecognized as *home*, since *lying down* at *home* and with a *neutral* emotional state is identified as the high-level context *sleeping*. Moreover, errors simultaneously present in various low-level contexts generally increase the chance of misidentification of the actual high-level context. Therefore, the combinations of errors in several low-level categories report a lower accuracy in the high-level context recognition than in the case of having only errors in a single category. As it was expected, the highest impact is observed when all three low-level contexts are subject to error. Either way, the error in the recognition of the high-level context remains below the level of error introduced in the considered low-level contexts. Finally, it must be noted that owing to the descriptive logic characteristic of ontologies, and conversely to probabilistic classification models, combinations of correct low-level contexts will always lead to a correctly inferred high-level context.

### 5.2. Performance of the Mining Minds High-Level Context Architecture

The HLCA has been implemented and benchmarked in order to assess its performance. The HLCA has been implemented in Java using available open source libraries. All the components of the HLCA build on Apache Jena (v2.11.2) [40], a semantic web framework which includes some APIs for handling RDF [41], OWL [34], and SPARQL [39]. In the implementation of the High-Level Context Reasoner, an off-the-shelf open source reasoner, namely Pellet (v2.3.2) [38], has been utilized in combination with Jena to enable the ontological inference functionalities. Furthermore, in the Context Manager, the Jena Triple Store (TDB) has been used as the Context Storage for the persistence of the Mining Minds Context Ontology. The communication between the Low-Level Context Architecture and the HLCA has been implemented by means of RESTful web services [42] and establishing service contracts among these two architectures. The same mechanism applies to the communication of the HLCA with the third party applications registered to get access to the newly identified high-level contexts.

For the evaluation, this implementation of the HLCA has been executed on a laptop operating Windows 10 with a 1.80 GHz Intel Core i7 CPU, 8GB RAM, and a HDD with 5400-RPM spindle speed, I/O data-transfer rate up to 6 Gb/s and 16 MB buffer. Using a test Java application the Low-Level Context Architecture has been emulated. The evaluation has consisted in the generation of 250,000 random low-level contexts belonging to 100 different users and which represented their context information for a time span of 16 days. First the category of the low-level context (activity, location or emotion) has been randomly selected and then one of the types for that category has also been randomly chosen. After that, the metadata associated to the low-level context label has been generated. The low-level context has been randomly assigned to one of the 100 users. The start time of each low-level context has also been randomly selected between 1 and 10 s after the start time of the previous low-level context. The generated low-level contexts, including the labels and the metadata, have been input one at a time to the HLCA for their mapping, synchronization, instantiation, verification, classification and notification. It is important to notice that the low-level contexts are served to the HLCA sequentially and at their simulated occurrence time. Thus, the HLCA works at real-time and processes each single instance on-the-fly right after receiving it. Concurrency is procured through user-based multithreading, thus supporting simultaneous processing of low-level contexts from different users taking place at the same time. Some resources such as the Context Storage are shared among threads (users). During the evaluation the time required for the context identification has been calculated and the volume of information generated and stored on the Context Storage has further been determined.

**Figure 9:**
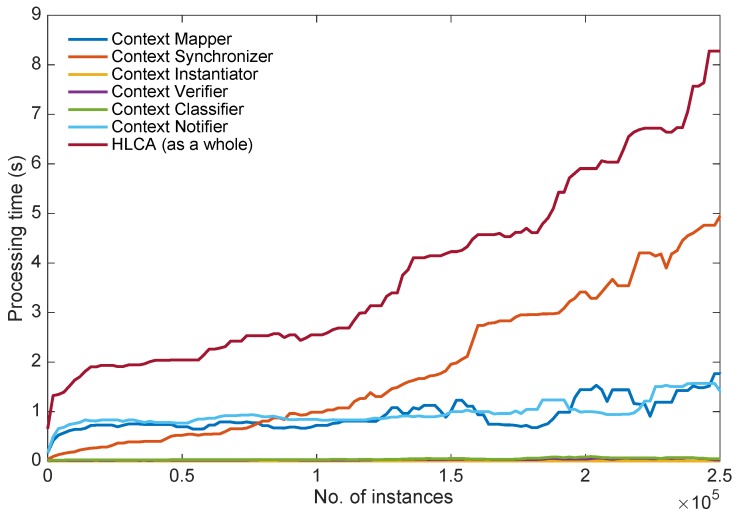
Processing time invested by each of the HLCA components in the context identification. The number of instances indicates the amount of previously processed high-level contexts when the recognition process is triggered.

Figure 9 shows the time invested by each of the HLCA components and the system as a whole in the context identification process. The number of instances indicates the number of high-level contexts which have already been processed by the HLCA when the recognition process is triggered due to a change in the low-level context. Even if the context recognition process is performed instance-wise, the number of previously processed instances is important because of the volume of information generated by the system during the recognition process and persisted in the Context Storage. The processing times are further averaged to have an overall figure summarizing the time taken by the each component of the HLCA. Table 2 presents the mean and standard deviation of these times as well as the percentage of these times devoted to the interaction of the component with the Context Manager. This interaction is particularly relevant because the Context Manager hosts the Context Storage, the shared resource which persists and loads the context information.

One can observe the differences of scale in the processing times for each of the components of the HLCA and the disparate tendencies of these times when the number of recognized context instances increases. The processes in which the HLCA component does not have any interaction with the Context Storage take much less time than the ones involving it. Furthermore, in the cases where the Context Storage is not involved, the processing time does not increase with the number of identified context instances. The Context Classifier and the Context Verifier take only some milliseconds to verify and classify the high-level context instance. This time is quite small due to the architectural design principle for which each single instance of high-level context is reasoned separately on-the fly at run-time. The Context Instantiator does not access either the Context Storage, since the required interactions to find the concurrent low-level contexts are performed by the Context Synchronizer. Therefore, the Context Instantiator takes only one millisecond to create a new instance of high-level context and this time does not increase with the number of instances because of the independence of the process from any other high-level context.

In case the components of the HLCA invoke the Context Storage, the processing times rise and the interactions with the Context Storage tend to represent most of the computational time, specifically more than 99%. This means that the actual component is relatively quick to perform its job but the context read and write processes which involve the Context Manager delay the complete process. The processing time for the Context Mapper and the High-Level Context Notifier follow a similar pattern. These processing times increase with the number of instances, at the beginning and with very few instances the times rocket, but then they stabilize and reach values around one second. The similarity in the evolution of the processing times for these two components is normal because their interactions with the Context Manager are of the same type. In the first case, the Context Mapper stores the new low-level context instance, retrieves the previous low-level context and after updating it, stores it again into the Context Manager. In the second case, the High-Level Context Notifier stores the new high-level context instance, retrieves the previous high-level context, compares them and after updating the previous instance, stores it again into the Context Manager. Therefore, the evolution of the processing time for operations that involve read and write to the Context Manager can be observed in the times for the Context Mapper and the High-Level Context Notifier. The processing performed by the Context Synchronizer in order to request concurrent low-level context instances is the most time demanding process of the HLCA. In this case, most of the time is devoted to the execution of the SPARQL queries and the retrieval of the matching solutions from the Context Manager. The processing time for the Context Synchronizer increases almost lineally with the number of instances. In fact, for few instances this time is below the processing time for the Context Mapper and High-Level Context Notifier, but then it becomes much higher. Therefore, the Context Synchronizer is the bottle neck of the HLCA, with a clear impact on the evolution of the time required for the context identification.

The relevance of the time invested by the HCLA to recognize a high-level context fairly depends on the application domain. Thus for example, if an alert has to be sent right away or a prompt action be taken based on the detected high-level context, then this time might be arguably long. However, if the identified information is rather used for analyzing the trajectories of behavior over time, then this time turns to be hardly relevant. Under these considerations, the processing time for the recognition of high-level contexts could be the main limitation of the actual implementation of the HLCA and should be improved in future work. A potential solution could consist in introducing a cache system into the High-Level Context Builder to save temporarily only the latest instances of low-level context and periodically persist them into the Context Manager. With such a solution the Context Synchronizer would not need to interact with the Context Manager and could pose the SPARQL queries directly to the cache; thus, retrieving the low-level context instances from a much smaller store. The Context Mapper, also part of the High-Level Context Builder, could share this very cache with the Context Synchronizer and increase its performance as well. If the cache would prove to be a good solution, such a system could also be introduced in the Context Notifier. This component has a similar behavior than the Context Mapper and its processing time could be reduced as well. Alternate solutions for accelerating the processing time for the identification of high-level contexts could include parallelizing tasks, defining different levels of cache-memory or simply scaling the infrastructure through cloud-based services.

**Figure 10:**
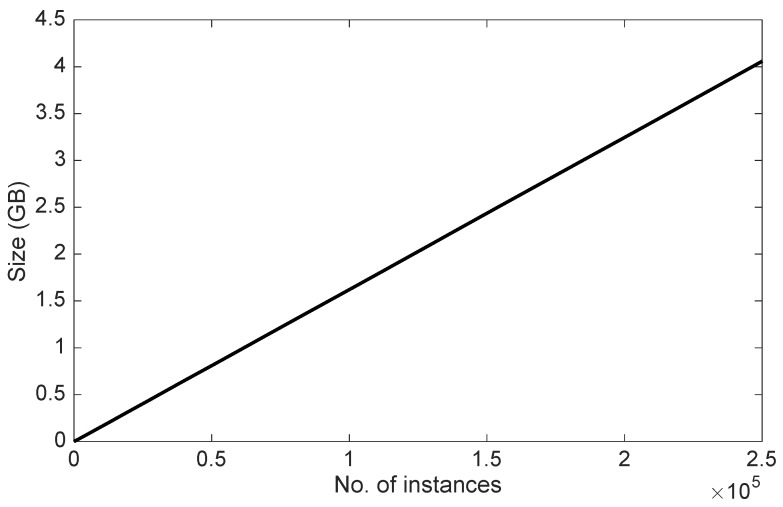
Size of the Context Storage depending on the number of persisted instances of high-level context. It must be noted that the storage of each high-level context instance has associated the storage of the low-level context instance which triggered its creation. Thus, for example, 250,000 instances in the X-axis represent 250,000 high-level contexts plus 250,000 low-level contexts stored on disc.

Finally, Figure 10 depicts the size of the Context Storage in the Context Manager increasing lineally with the number of stored high-level context instances. The initialization of the Context Storage, i.e., storing the terminology defining the Mining Minds Context Ontology, requires only 408.5 KB on disc. The storage of each new high-level context instance, which has associated the storage of the low-level context instance which triggered its creation, increases the size of the Context Storage in 17 KB, in average. Thus, for the previous simulation of 250,000 changes in the context, which leads to a total of 500,000 context instances on disc (i.e., 250,000 high-level context instances and 250,000 low-level contexts instances), the Context Storage reached a size of 4.06 GB. Despite the Context Manager proves to fairly handle this volume of data, the increasing time observed for I/O operations in long-term scenarios with several users demands for some of the aforementioned solutions.

## 6. Conclusions

This work has presented an ontology-based method for deriving high-level context information out of the combination of cross-domain low-level context primitives, namely activities, locations and emotions. The modeling of the low and high-level contexts is defined through the so-called Mining Minds Context Ontology, while the processing and inference of contexts is performed by the Mining Minds High-Level Context Architecture, both contributions of this work. The Mining Minds Context Ontology has been designed to support any combination of low-level contexts to define a specific high-level context. The unprecedented incorporation of emotions in the context definition enables the representation of new high-level contexts that can only be identified whenever a specific emotion takes place. The Mining Minds Context Ontology has also been designed to procure the identification of some high-level contexts even in the absence of emotion information. The Mining Minds High-Level Context Architecture builds on the Mining Minds Context Ontology and reasoning techniques to enable the inference of high-level context from low-level context primitives in real time. The evaluation of the implemented architecture proves the reasonably good robustness properties of the Mining Minds Context Ontology against potentially erroneous low-level contexts. In fact, the results have showed that the impact of the error introduced in the low-level context is always lower at the high-level and that the activity is the most prevalent category in terms of error impact, while the location and especially the emotion tend to show a lesser effect on the high-level context. The current prototype implementation of the Mining Minds High-Level Context Architecture has been proven to perform well with respect to processing time and management of context instances. However, in order to ensure the scalability of the Mining Minds High-Level Context Architecture, the synchronization and database transactions management needs to be improved. Future work includes modifications in the database management to accelerate the inference time, the evaluation of this architecture with real users and the evolution of the Mining Minds Context Ontology in order to include more types of low-level context and new identifiable high-level contexts.

## Figures and Tables

**Table 1:** Mean and standard deviation of the accuracy of the high-level context recognition under different levels of errors in the detected low-level contexts.

	5%	10%	20%	50%
**Activity**	97.60 ± 0.05	95.13 ± 0.05	90.39 ± 0.04	75.32 ± 0.20
**Location**	99.45 ± 0.02	98.82 ± 0.05	97.61 ± 0.15	93.93 ± 0.02
**Emotion**	99.63 ± 0.02	99.18 ± 0.05	98.32 ± 0.05	96.04 ± 0.07
**Act & Loc**	97.08 ± 0.10	94.27 ± 0.16	88.48 ± 0.11	72.63 ± 0.10
**Act & Emo**	97.16 ± 0.12	94.22 ± 0.06	89.60 ± 0.10	73.53 ± 0.30
**Loc & Emo**	99.00 ± 0.05	98.02 ± 0.09	96.24 ± 0.05	91.25 ± 0.09
**Act & Loc & Emo**	96.56 ± 0.06	93.10 ± 0.30	87.52 ± 0.11	71.60 ± 0.13

**Table 2:** Mean and standard deviation of the processing time invested by each of the HLCA components in the context identification, as well as the percentage of these times devoted to the interaction with the Context Manager.

	Context Mapper	Context Synchronizer	Context Instantiator	Context Verifier	Context Classifier	Context Notifier
**Mean (s)**	0.986	2.188	0.001	0.032	0.046	1.012
**Standard Deviation (s)**	0.348	1.670	0.000	0.014	0.019	0.268
**Context Manager (%)**	99.53	99.97	0.00	0.00	0.00	99.99
